# Thymic Epithelial Tumors: An Evolving Field

**DOI:** 10.3390/life13020314

**Published:** 2023-01-22

**Authors:** Elisabetta Kuhn, Carlo Pescia, Paolo Mendogni, Mario Nosotti, Stefano Ferrero

**Affiliations:** 1 S.C. Anatomia Patologica, Fondazione IRCCS Ca’ Granda Ospedale Maggiore Policlinico, 20122 Milano, Italy; 2 Dipartimento di Scienze Biomediche, Chirurgiche ed Odontoiatriche, Università degli Studi di Milano, 20122 Milano, Italy; 3 S.C. Chirurgia Toracica e Trapianti di Polmone, Fondazione IRCCS Ca’ Granda Ospedale Maggiore Policlinico, 20122 Milano, Italy; 4 Dipartimento di Patofisiologia Medico-Chirurgica e dei Trapianti, Università degli Studi di Milano, 20122 Milano, Italy

**Keywords:** thymoma, thymic epithelial tumors, thymic carcinoma, history, classification, staging, therapy

## Abstract

Despite their rarity, thymic epithelial tumors (TETs) have attracted much interest over the years, leading to an impressive number of histological and staging classifications. At present, TETs are divided by the WHO classification into four main subtypes: type A, type AB, and type B thymomas (subdivided into B1, B2, and B3), and thymic carcinomas, going from the more indolent to the most aggressive ones. Among many debated staging proposals, the TNM and the Masaoka–Koga staging systems have been widely accepted and used in routine practice. The four-tiered histological classification is symmetrically mirrored by the molecular subgrouping of TETs, which identifies an A-like and an AB-like cluster, with frequent *GTF2I* and *HRAS* mutations; an intermediate B-like cluster, with a T-cell signaling profile; and a carcinoma-like cluster comprising thymic carcinomas with frequent *CDKN2A* and *TP53* alterations and a high tumor molecular burden. Molecular investigations have opened the way to tailored therapies, such as tyrosine kinase inhibitors targeting *KIT*, mTOR, and *VEGFR*, and immune-checkpoints that have been adopted as second-line systemic treatments. In this review, we discuss the crucial events that led to the current understanding of TETs, while disclosing the next steps in this intriguing field.

## 1. Introduction

Thymic epithelial tumors (TETs), although rare, are the most frequent neoplasias in the anterior mediastinum and occasionally occur ectopically, mainly in the neck. Altogether they have an incidence of 0.23–0.30 cases per 100,000 people per year, with a peak between the 4th and 6th decades [1,2]. TETs constitute a unique spectrum of neoplasias that include thymoma, thymic carcinoma, and thymic neuroendocrine neoplasms. Characteristically, thymomas display lobular growth, an intratumoral component of immature T lymphocytes, and perivascular spaces, which represent the architectural features that recapitulate the organoid formation of the thymus. Clinically, TETs are asymptomatic (one-third of cases) or manifest with mass effect symptoms, autoimmune diseases such as myasthenia gravis (MG) and peripheral neuropathy, and immunodeficiencies such as lymphopenia and hypogammaglobulinemia, among others [3].

For decades, the classification of TETs has been elusive and controversial, until the World Health Organization (WHO) Committee, led by Juan Rosai, proposed in 1999 a simplified classification using a combination of letters and numbers that is still in use, with only minor modifications, and widely accepted [2,4].

Similarly, several TET staging systems have been proposed and revised during the last 50 years, leading to much confusion about the most appropriate system and about which carries the best prognostication ability. Local invasiveness, at least in thymomas, plays a pivotal role in determining the disease stage, as distant hematogenous and lymphatic spread is uncommon. At present, TNM and Masaoka–Koga stagings are the most widely used, with the first set as mandatory by the last WHO classification, and the second as optional [2].

The molecular landscape of TETs has started to be delineated only in recent years, when The Cancer Genome Atlas (TCGA) performed a multiplatform-integrated next-generation sequencing (NGS) study on TETs [5,6]. Based on clustering analysis, the TCGA could divide TETs into four main subtypes that mirror the WHO histological classification. Subtype 1 encloses type B thymomas, heavily enriched with tumors associated with MG. Subtype 2 defines the aggressive subgroup of thymic carcinomas, bearing a higher tumor mutational burden (TMB), upregulation of oncogenes, downregulation of tumor suppressors, and frequent chromosome 16q loss. Subtype 3 comprises type AB thymomas, with a high prevalence of *GTF2I* mutations and rich lymphocytic components. Subtype 4 is constituted by type A and type B thymomas, with a high prevalence of *GTF2I* and *HRAS* somatic mutations. Unsurprisingly, thymic carcinomas harbor the highest molecular complexity among TETs, showing common alterations in *CDKN2A*, *TP53*, and *ZNF429*, and less frequently *KIT*, *PTEN/PI3K/mTOR*, *ATM*, *ALK*, *ERBB2*, and *CYLD1* genetic abnormalities [5,6,7].

Although, the suggested first-line therapeutic approach to TETs is represented by surgery, in advanced, metastatic, locally aggressive or inoperable cases, radiotherapy and systemic chemotherapy hold important roles as ancillary tools. Specifically, first-line systemic therapy is mainly represented by platinum-based chemotherapy. Furthermore, targeted therapy has recently shown promising potential in TETs, especially in thymic carcinoma, where KIT inhibitors, VEGFR/multikinase inhibitors (such as sunitinib), and mTOR inhibitors (such as everolimus) have been introduced as second-line treatments for cases refractory to first-line chemotherapy [8,9]. Likewise, several trials have proven immune-checkpoint inhibitors (i.e., pembrolizumab, avelumab) to be effective in TETs. Nevertheless, these drugs must be used with caution in TETs, as a considerable percentage (>15%) of cases develop autoimmune complications, which are sometimes even fatal [10,11].

In the current manuscript, we review the state-of-the-art in the TET field, starting from a historical overview of the classification and staging, continuing with the current knowledge of TET molecular characterization and standard therapeutic practice, and eventually outlining the future perspectives of tailored therapy.

## 2. Histopathological Classification of Thymic Epithelial Tumors

### 2.1. Historical Background

**Figure 1:**
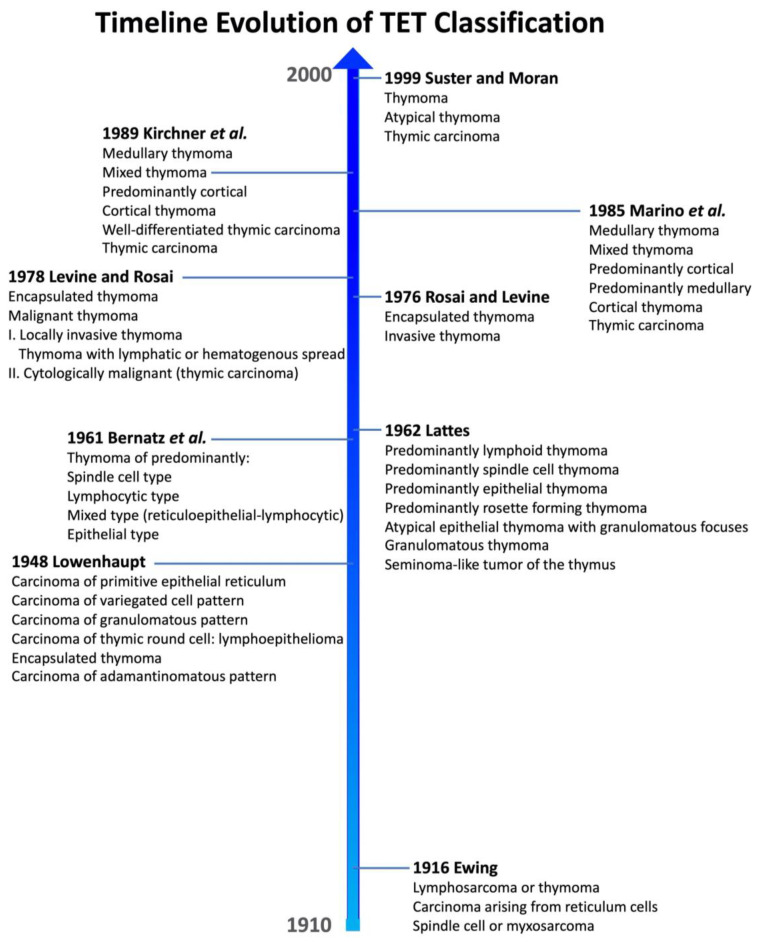
Timeline of thymic epithelial tumor histological classification.

For almost a century, the histopathological classification of TETs has challenged the pathologist community (Figure 1). There are many reasons behind this inscrutability: (1) the rarity of this pathology; (2) the mixture of lymphoid and epithelial cell populations in various proportions, which produces a remarkable morphological heterogeneity; (3) a long-lasting misperception of which cell component is truly neoplastic; (4) the large histogenetic variety of thymic-located tumors; and (5) the debatable prognostic role of different TETs. Initially, the TET classifications suffered from complete ignorance regarding the normal architecture and function of the thymus gland, as well as its embryologic development.

The first classification proposed by James Ewing in 1916 (and subsequently widely used) adopted the nowadays ambiguous nomenclature of “lymphosarcoma or thymomas” to designate a heterogenous group of tumors that likely combine certain types of thymic lymphoma together with true thymomas [12].

In 1948, Lowenhaupt proposed an innovative classification, which was rooted in brand-new embryologic concepts that were emerging but introduced novel terms that were further never used in the field [13,14]. Nevertheless, she univocally stated the epithelial derivation of TETs and indicated complete surgical excision as the treatment of choice [14].

In 1961, Bernatz et al. proposed a TET morphologic classification based on both the shape of epithelial cells and the proportion between lymphocytes and epithelial cells [15]. These authors distinguished four thymoma categories: predominantly lymphocytic, predominantly epithelial, predominantly mixed, and predominantly spindle cell. This classification scheme gained popularity in the United States and was applied for at least two decades. In the following year (1962), Lattes proposed a morphological classification that kept into account, other than the lymphocytic content and the epithelial cell type, also the presence of tumor encapsulation and invasion as fundamental features for the differential diagnosis between benign and malignant thymomas [16]. Lattes was persuaded of the epithelial origin of these tumors and that the thymoma subtyping by itself could not be sufficient to correctly predict TET clinical behavior.

Subsequently, Rosai and Levine proposed in 1976 to limit the designation of “thymoma” to tumors of thymic epithelial derivation, regardless of the presence of a thymocyte component, ruling out once and for all other thymic tumors to be classified according to the cell of origin, such as carcinoids, germ cell tumors, and malignant lymphomas [17]. Their classification was a simplified two-tiered system that distinguished encapsulated from invasive thymomas. This proposal was motivated by the difficulty of enclosing the whole continuous range of morphological variety of thymomas in rigid criteria. Two years later, the same authors refined their classification, distinguishing between benign encapsulated thymomas and malignant thymomas. The latter were further divided into invasive thymomas (type I), either locally or with lymphatic or hematogenous spread, and cytologically malignant thymomas (type II) [18], thus including thymic carcinoma in the classification. This classification was widely accepted and applied for many years.

In 1985, Muller-Hermelink and collaborators proposed a new approach to TET classification, which attempted to show the correlation between TET neoplastic cells and their normal compartment of likely origin in the thymus gland, recognizing in this way the following subtypes: medullary, mixed, predominantly medullary, predominantly cortical, and cortical thymomas and thymic carcinoma [19]. A few years later, this classification was updated with the introduction of “well-differentiated thymic carcinoma”, a carcinoma that maintains at least a focal organotypic differentiation in the form of lobular architecture or perivascular spaces [20]. This classification is known as the histogenetic or European classification.

Suster and Moran proposed in 1999 a drastically simplified classification of TETs, founded on the premise that TETs are a morphological continuum from well-differentiated to poorly differentiated [21]. They recognized three diagnostic categories: thymoma, atypical thymoma, and thymic carcinoma. The degree of differentiation depended on the degree of cytological atypia and the presence of thymic organotypic features.

Several other classifications were proposed in the last century that are not mentioned here for the sake of brevity [22].

### 2.2. The WHO Classification

#### 2.2.1. The Way to Current WHO Classification

Only in 1999 did the WHO present the first international collaborative effort to unify the plethora of TET classifications in a unique shared histological classification scheme [4]. A WHO Committee, composed of 10 pathologists from all around the world and led by Juan Rosai, published a simplified classification using a combination of letters and numbers (Table 1). This classification with the capital letter A for atrophic, B for bioactive, and C for carcinoma refers to epithelial cell shapes specific to different subtypes: spindle/medullary for letter A, plump and dendritic (cortical) for letter B, and overtly atypical for letter C. Moreover, the B subtype was further subclassified in B1, B2, and B3 according to the decreasing proportion of lymphocytic infiltrate. Therefore, this classification distinguished six types of thymoma (thymoma type A, AB, B1, B2, and B3; and type C thymoma or thymic carcinoma) based on cytoarchitectural features and independently from the staging, which instead was emphasized in the previous Rosai classification [17]. Subsequently, several studies confirmed the prognostic relevance of the 1999 WHO classification, further validating it [23].

The WHO classification has been revised three times so far (Table 1). Firstly, in 2004, “type C thymoma” terminology was abandoned in favor of the more univocal term “thymic carcinoma”, and the classification was enriched with clinical aspects, prognostic data, immunohistochemical features, and genetic information [24]. The following 2015 classification, on one hand, implemented an interdisciplinary approach to TETs with the contribution of radiologists, thoracic surgeons, and oncologists, and on the other refined the epidemiologic, imaging, cytologic, and prognostic data from the International Thymic Malignancy Interest Group (ITMIG), a centralized retrospective database that comprises over 6000 cases worldwide [25,26]. This classification maintained the previous scheme and refined the cytoarchitectural and immunohistochemical criteria in order to improve the diagnostic reproducibility of TET subtypes [25]. Notably, an atypical type A variant was added, where “atypia” refers to the presence of hypercellularity, increased mitotic activity, and necrosis in an otherwise normal-appearing type A thymoma. Moreover, NUT carcinoma was included as a subtype of thymic carcinoma [24]. Finally, the 2015 WHO classification recommended for the first time the diagnosing of heterogenous thymomas by recording all subtypes represented in the surgical specimen as 10% increments.

#### 2.2.2. Current 2021 WHO Classification

The latest edition of the WHO classification of Thoracic Tumors was released in 2021 [2]. As for TETs, it mainly represents a revision of the previous fourth edition of 2015, maintaining the same conceptual scheme and nomenclature introduced in 1999. Two variants of thymomas were removed, that is, microscopic and sclerosing thymomas, now considered, respectively, nodular epithelial hyperplasia, without documented malignant potential, and a regressive feature of a conventional thymoma.

Three new rare subtypes of thymic carcinoma were included:

1. Micronodular thymic carcinoma with lymphoid hyperplasia, the malignant counterpart of micronodular thymoma with lymphoid stroma;
2. Hyalinizing clear cell carcinoma, analogous to that from the salivary glands;
3. Thymic sebaceous carcinoma, similar to the homonym tumor of the skin.

Moreover, the nomenclature of some thymic carcinoma subtypes was refined. Papillary adenocarcinoma has become low-grade papillary carcinoma, whereas high-grade carcinoma with papillary features is included within adenocarcinomas NOS. Similarly, mucinous carcinomas without immunohistochemical expression of enteric markers (CK20, CDX2, MUC2) are now classified as adenocarcinomas NOS. Lastly, the term lymphoepithelial carcinoma is preferred to lymphoepithelial-like carcinoma.

Importantly, the reporting recommendations for histologically heterogeneous thymomas were confirmed and also integrated with comprehensive indications regarding the diagnostic reporting of combined TETs, in particular taking into account the aggressiveness of the single components over the percentage.

Finally, the recent WHO classification was further strengthened by novel genetic findings, in particular the identification of specific chromosomal translocation as molecular drivers of distinct TET subtypes (see below).

## 3. Staging Systems

### 3.1. Historical Background

Over the years, several authors have proposed many different clinico-pathological staging systems for TETs, and at least four different staging systems are currently used across the world (TNM, Masaoka, Masaoka-Koga, and Groupe d’Etude des Tumeurs Thymique [GETT]) [27,28]. Given that a detailed historical review of all the different staging systems is not our purpose, we will offer a simple perspective on the current understanding of TET staging and future directions.

Since the first staging system proposed by Bergh et al. [29], the local invasiveness of TETs has been shown to play a key role in defining the clinical outcome of these neoplasias, much more than the lymphatic and hematogenous spread of disease. In 1981, Masaoka et al. [30] proposed a four-tiered staging system that stressed the prognostic value of tumor local invasiveness based on their observation that TETs were slowly growing tumors with low rates of distant spread.

In parallel, after the first attempts to formulate a Tumor-Node-Metastasis staging system for TETs incorporating Masaoka stages [31,32,33], a proper TNM classification was implemented, and its most recent update is included in the 8th edition of the TNM classification and in the latest WHO thoracic tumor blue book [2].

The GETT staging system, published in 1982 and still used by some institutions in France [34], combined the extent of invasion with the completeness of resection. However, despite complete resection remaining one of the best prognostic factors, most authors disregard its inclusion in the staging classification and prefer it to be designated separately [35].

### 3.2. Main Staging Systems

#### 3.2.1. TNM Staging

The current TNM staging is the product of a fruitful partnership between the International Association for the Study of Lung Cancer (IASLC) and the ITMIG [36,37,38]. In this scheme (Table 2), microscopic confirmation is considered essential for correct pathological staging, overcoming the problem of macroscopic-based definitions. Moreover, the current TNM classification does not distinguish between encapsulated TETs and TETs invading the surrounding adipose tissue, because outcome findings did not demonstrate any difference between these two groups (corresponding to Masaoka–Koga stages I and II). Of note, TNM staging is particularly indicated, compared to Masaoka–Koga staging, in the evaluation of thymic carcinomas, which frequently exhibit lymphogenous (25%) or hematogenous (12%) spread [27].

Nonetheless, despite the number of efforts to make TET staging simple, understandable, and practical, there are still many gray zones, especially in the evaluation of the pathological T stage in routine “real-life” settings, as highlighted by recent studies [28,39]. A debated question is whether to include tumor size in the T category or not, since several studies have reported tumor size to be prognostically relevant [40,41,42]. However, in the IASLC/ITMIG validation cohort, tumor size was prognostically significant only in incomplete resections and advanced stages. Therefore, it was not included in the 8^th^ edition of the TNM classification. In another study, tumor size was only predictive of increased risk of disease recurrence [43]. Other authors have reported a putative prognostic role for tumor size, but only in selected histological subtypes, with different, nonstandardized threshold sizes, and sometimes even in the presence of potential confounders [44,45,46]. Therefore, a wise suggestion would be to always report the size of the tumor in the pathological report while we wait for future studies that will clarify such matters.

#### 3.2.2. Masaoka–Koga Staging

The original Masaoka staging system was subsequently modified by Koga et al. in 1994 [47], becoming the four-tiered Masaoka–Koga modified system that currently is the most widely used among TETs [48] (Table 2).

Interestingly, due to the lack of statistically significant differences in outcomes between stages I and II and between stages III and IV, a two-tiered system distinguishing invasive from not invasive cases is likely to be suitable as well [27,35,49].

In 2011, the ITMIG officially adopted the Masaoka–Koga staging system, identifying problematic terminologies used in this staging that had led to variable interpretations, such as the meaning of “transcapsular invasion” or the differences between “adherence to” and “invasion of” the mediastinal pleura or pericardium [50]. Thus, the ITMIG published the “clarification and definition of terms” document, which also dealt with other common problems within TET staging. For instance, a partially unencapsulated thymoma should not be interpreted as an “invasive” thymoma (at least stage II according to Masaoka–Koga) but rather as a stage I disease. Stage IIb disease should also be confirmed microscopically, as a histologically proven invasion should prevail over any macroscopic impression. Moreover, the ITMIG proposed the Masaoka–Koga staging system as suitable also for carcinomas of the thymus, including thymic neuroendocrine tumors and other less common histological subtypes.

#### 3.2.3. Comparison between the TNM and Masaoka–Koga Staging Systems

According to the TNM staging, Masaoka–Koga stages I, II a, and II b are all comprised within the T1a definition, while stage III cases are classified as T1b if they invade the mediastinal pleura; as T2 if they invade the pericardium; as T3 if they invade the lung, the brachiocephalic vein, superior vena cava, phrenic nerve, chest wall, extrapericardial pulmonary artery, or vein; or as T4 if they invade the aorta, arch vessels, intrapericardial pulmonary artery, myocardium, trachea, and esophagus.

Lymph node metastases are exceedingly rare in thymomas and therefore were not considered in the Masoka–Koga staging system. In the TNM classification, the N category is defined by the presence of perithymic lymph node involvement (N1) or deep thoracic or cervical lymph node involvement (N2).

Lastly, the M category is defined either by pleural or pericardial metastasis (M1a), corresponding to Masaoka–Koga stage IV a, or pulmonary intraparenchymal or distant metastasis (M1b), corresponding to Masaoka–Koga stage IV b.

### 3.3. Prognostic Factors

The main clinico-pathological prognostic factors for TETs are tumor size, spread, resection status, histological type, patient characteristics (age and comorbidities), and treatment response [43,51,52,53]. In addition, few studies have reported the prognostic value of serum biomarkers, in particular lactate dehydrogenase and NSE, neutrophil-to-lymphocyte ratio, and immunohistochemical stainings, such as bcl-2, ki-67, MMP-2, p21, and TIMP-2 [54,55,56,57,58,59,60].

## 4. Molecular Pathology

The molecular landscape of TETs has been poorly characterized until recently, when more rapid, sophisticated, and sensitive techniques, such as NGS, provided us with higher-quality molecular data, allowing for the simultaneous identification of many genetic alterations together with the TMB [6,61]. Some of the reasons for this delay are strictly related to TET rarity and their morphological characteristics, in particular the rich component of non-neoplastic thymocytes intermingled within the more common entities (i.e., type AB, B1, and B2 thymomas). In fact, the relative abundance of non-neoplastic cells hampers the application of many molecular methods, such as array-based comparative genomic hybridization (CGH) and fluorescence in situ hybridization (FISH) [62]. Notably, in recent years the rise of targeted therapies for human cancers has represented a major drive for better molecular profiling of tumors, including aggressive cancers and rare entities, such as TETs [6].

One of the earliest notions acquired regarding TET molecular alterations has been the higher frequency of large copy number variations (CNVs) involving entire chromosomes among thymic carcinomas and B2 and B3 thymomas compared to more indolent TETs (such as type A and type AB thymomas) [62,63]. These CNVs are either represented by losses in chromosomes 6, 3p, 16q, and 13q, or gains in chromosomes 1q, 7, and 20p. Amplification of the *BCL2* gene has also been observed and linked to its overexpression, as proved by RNA-seq investigations [63]. In this regard, array-CGH studies found that gains in the *BCL2* gene, as well as *CDKN2A* losses (due to chromosome 16q loss), were associated with poorer outcomes among TETs [64]. Immunohistochemically, p16 loss parallels *CDKN2A* deletion [64].

DNA hypermethylation resulting in gene silencing may also play a role in TETs, involving various targets such as *CDH1*, *CDKN2A*, *FHIT*, *MGMT*, and *MLH1*. *MGMT* methylation, for example, has been found more frequently in thymic carcinoma, and it is linked to a worse stage at diagnosis as well as to a better response to alkylating agents [65,66].

**Figure 2:**
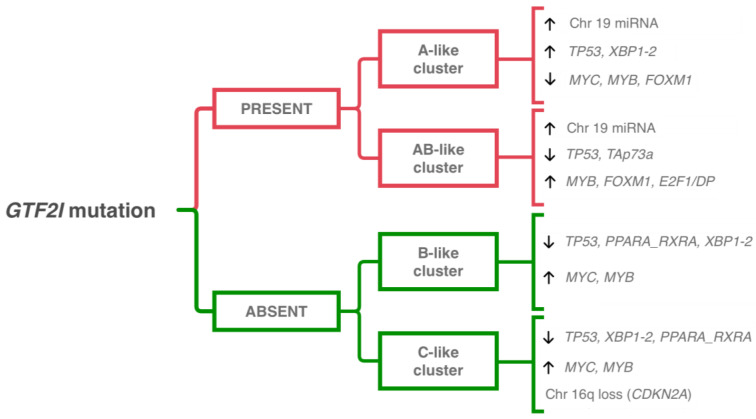
Integrative clustering combined with single and multiplatform analyses results in four different groups of TETs, mirroring the current histopathological classification [5]. Abbreviations: Chr, chromosome.

Recent multiplatform analyses applying NGS techniques have provided a more detailed molecular characterization and subclassification of TETs. The TCGA project [5] has performed a thorough molecular subclassification of TETs, which mirrors the actual WHO histological classification, proving that histologically different TETs are effectively biologically distinct and not part of a disease continuum. Through integrated clustering analysis of whole genome sequencing results, TETs can be divided into four groups: (a) subtype 1, comprising type B thymomas; (b) subtype 2, comprising thymic carcinoma; (c) subtype 3, comprising mostly type AB thymomas; and (d) subtype 4, represented by a mix of type A and type B thymomas. Subtypes 1 and 3 are enriched in lymphocytes, subtypes 3 and 4 are predominantly defined by *GTF2I* mutation, and subtype 4 harbors frequent *HRAS* mutation. As expected, subtype 2 proved to be associated with poorer overall survival compared to the other subtypes. Moreover, using a TumorMap approach, it was possible to expand the four-tiered clustering of TETs (Figure 2): type A and AB thymoma clusters were defined by *GTF2I* mutations, and the overexpression of a large miRNA cluster on chromosome 19q13.42 activating the *PI3K/mTOR* pathway, while type C cluster (i.e., thymic carcinomas) was defined by chromosome 16q loss. Single and multiplatform analyses identified the upregulation of tumor suppressor genes (i.e., *CDKN2A* and *TP53*) and downregulation of oncogenes (i.e., *FOXM1, MYB*, and *MYC*) in the type A cluster, while the opposite was observed in type AB, B, and C clusters.

At a later time, Lee et al. [67] confirmed, using the TET TCGA dataset, the molecular subclassification in four groups, although with some minor differences. Specifically, the four groups are divided as follows: the *GTF2I* mutated group, with the most favorable histology, lower Masaoka stage, and rare association with MG; the *GTF2I* wild-type group with a T-cell signaling profile (TS group); the *GTF2I* wild-type group with low CNVs (chromosomally stable group, CS); and the *GTF2I* wild-type group with chromosomal instability (chromosomal instability group, CIN), with the most aggressive histology, higher Masaoka stage, and more frequent association with MG.

What stands out about these deep molecular analyses is not only the almost exact correspondence between histological classification and molecular makeup, but also the pivotal role of the *GTF2I* mutation in TETs. Indeed, *GTF2I* is a thymoma-specific oncogene, unique to TETs and not found in other human cancers [63,67]. Remarkably, the *GTF2I* mutation occurs at a single codon (L424H) and has been found in almost 100% of type A thymomas and in about 70% of type AB thymomas [5], while its prevalence decreases in more aggressive TETs, being found only in 8% of thymic carcinomas. Accordingly, *GTF2I* mutated TETs have shown statistically significant better prognoses compared to *GTF2I* wild-type cases [63]. Functionally, *GTF2I* encodes for TFII-I protein, which is involved in the transcriptional regulation of genes implied in the cell cycle, DNA repair, cell proliferation, and *TSC/mTOR* signaling [63,68].

By means of bioinformatic analysis of the TCGA database, TETs have revealed 134 genes that were silenced by promoter hypermethylation, with 174 upregulated miRNAs [69,70]. Specifically, *CDKN2A* promoter methylation has been observed in thymomas and thymic carcinomas, presumably leading to the inactivation of the p16/RB axis [71], while higher levels of *MTHFR* methylation were observed in MG-associated TETs [72]. Notably, genes are differentially methylated among TETs, and the methylation levels of specific sites might hold a prognostic significance. DNA methylation is in fact more frequent in thymic carcinoma compared to thymoma, in such a way that the methylation frequency in TETs runs parallel to their malignant behavior [73,74]. Moreover, TETs show frequent alterations in non-coding RNAs, such as miRNA and long non-coding RNAs. As previously said, the large C19MC miRNA cluster has a significant association with type A and AB thymomas, while it might be silenced via promoter methylation in thymic carcinoma [6,74,75,76]. Additionally, an aberrant decrease in miRNA-19b has been observed in MG-associated TETs, contributing to a decrease in thymic stromal lymphopoietin (TSLP) and T-helper 17 cell development [77]. Of interest, in addition, differential expression of selected non-coding RNAs might be helpful in distinguishing TET subtypes and understanding their impact on different molecular pathways [78].

Growing evidence implies the oncogenetic role of chromosomal translocations in specific TETs. First, similarly to the corresponding tumors in other sites, the same pathognomonic gene fusions *CRTC1-MAML2*, *BRD4-NUTM1,* and *EWSR1-ATF1* have been detected in thymic mucoepidermoid, NUT, and hyalinizing clear cell carcinoma, respectively [79,80,81]. Then, in recent years, Massoth et al. [82] demonstrated the presence of *KMT2A-MAML2* gene fusion in up to 6% of type B2 and B3 thymomas, which is instead absent among type A, AB, B1 thymomas, and thymic carcinomas. This gene fusion might play an oncogenic role by disrupting *NOTCH1* signaling and could be targetable with epidermal growth factor inhibitors, as shown in *MAML*-fused mucoepidermoid carcinomas [83]. In addition, a distinct *YAP1-MAML2* translocation has been specifically identified in metaplastic thymoma [84].

TMB has recently gained much attention as a predictive marker of immune-checkpoint inhibitor response. Nevertheless, TETs have one of the lowest TMBs among human tumors, with an average of 1.92 mutations/Megabase among thymomas and an average of 3.84 mutations/Megabase in thymic carcinomas [5,7].

On the other hand, interesting studies suggest that autoimmunity symptoms (including MG), which are frequent in TET patients, might be the expression of antigenic diversity. Coherently, TCGA molecular subclassification highlighted a high prevalence of aneuploidy among MG-associated TETs, albeit no specific gene expression signature was identified [5]. In addition, MG-associated TETs revealed intratumoral overexpression of *NEF*, *CHRNA1,* and *RYR3* genes, which share sequence similarities with major autoimmune targets of MG. Accordingly, miRNA analyses demonstrated a high prevalence (80%) of *CCL25* gene overexpression among MG-associated TETs [85]. *CCL25,* located on chromosome 19, encodes for a chemokine associated with T-cell development, and its overexpression might be due to arm-long CNV. Interestingly, the same study was able to identify a two-fold increase in gene upregulation of the TGF-beta and HTLV-1 signaling pathways in MG-associated TETs, consistent with previous suggestions that HTLV-1 may play a role in MG pathogenesis [86].

Of note, the molecular landscape of thymic carcinoma alone is profoundly heterogeneous, and findings regarding the relative frequency of specific genetic alterations are not univocal. As mentioned above, thymic carcinomas bear the highest TMB among TETs, are *GTF2I* wild-type, and show upregulation of oncogenes and downregulation of oncosuppressors (mainly *TP53*). Furthermore, *BCL2* copy gain, *CDKN2A* copy loss (due to chromosome 16 CNV), and mutations in *TP53*, *ZNF429*, and epigenetic regulatory genes (i.e., *BAP1, ASXL1, TET2, DNMT3A,* etc.) are the most frequent genetic abnormalities in thymic carcinoma [6,87,88]. Of interest, *KIT* mutations, found in up to 11% of thymic carcinomas, represent one of the few targetable mutations in TETs [6,7,89]. In addition, *CYLD1* mutations, reported in more than 10% of thymic carcinomas, may play a putative role in promoting PD-L1 expression [90,91]. Other sporadic mutations can be observed in *ALK*, *ATM*, *ERBB2/3*, *EZH2*, *FGFR3, MET* genes, and PTEN/PI3K/mTOR pathways [7]. The prognostic relevance of all these genetic alterations is yet to be fully determined, as different authors have reported conflicting results [6,64,92]. Nonetheless, these mutations could be clinically relevant in the next future within the framework of personalized medicine, serving as therapeutic targets for this aggressive disease.

### TET Microenvironment

Several studies have focused on the tumor microenvironment of TETs, which is of particular complexity given the physiological role of the thymus gland within the immune system. The TET microenvironment overlaps largely with the normal thymus components. Predictably, thymomas show a higher proportion of lymphocytic infiltrate compared to thymic carcinomas; AB, B1, and B2 thymomas show a higher proportion of CD4+/CD8+ immature cells, while B3 thymomas and thymic carcinomas show numerous terminally differentiated CD4+ or CD8+ cells, mostly polarized toward a CD8+ cytotoxic phenotype [93]. Thymic carcinomas also show a lower expression of the pro-inflammatory gene *HMGB1,* portending a less favorable prognosis [94,95].

B-cells are particularly enriched in certain TET subtypes (micronodular thymoma or carcinoma with lymphoid hyperplasia) and in MG-associated TETs and other autoimmune disorders [93,96]. Lower-grade TETs, such as B1 and B2 thymomas, also show higher percentages of fascin and S100 positive dendritic cells compared to thymic carcinomas [97,98].

Other crucial tumor microenvironment components are tumor-associated macrophages (TAMs). TAMs are particularly enriched in TETs with higher TMB and in thymic carcinomas, where they are likely to exert pro-proliferative effects and facilitate tumor invasiveness [99,100]. TAMs within TETs express different heat shock proteins, such as HSP27 and HSP70, which contribute to tumor progression with their pro-inflammatory and anti-apoptotic functions. However, their immunohistochemical expression decreases gradually from type A thymomas to thymic carcinomas, while their serum concentration behaves oppositely, in contrast with other malignancies. Heat shock proteins might be particularly interesting as serum markers useful for disease monitoring and as immune therapeutic targets in the future, especially in combination with other regimens [101].

Lastly, the tumoral stroma could play a significant role in the TET microenvironment. For instance, fibronectin, a stromal protein diffusely expressed in cancer [102], has also been demonstrated in TET stromal cells.

## 5. Diagnostic and Therapeutic Approaches

The initial diagnosis of thymic neoplasms is usually suspected on a contrast-enhanced thorax computed tomography (CT) scan, which provides information about both the neoplasm and its relationship with surrounding structures. In addition, nuclear magnetic resonance is useful to differentiate between normal (or hyperplastic) thymic tissue and a thymoma or thymic carcinoma. Positron emission tomography (PET) is crucial in distinguishing between thymoma and thymic carcinoma, although there is little evidence about its role in differentiating hyperplasia from thymoma. Furthermore, the PET scan is a good predictor of TET histological classification [103].

The therapeutic standard for TET patients is still mainly represented by surgical resection of the neoplasm. Surgical techniques have undergone a continuous evolution over the years, in particular for early-stage neoplasm treatment. In the past, the standard approach to thymomas was basically an “open” surgery, with a total or partial sternotomy, cervicotomy, or both. With the development of minimally invasive thoracic surgery, the approach to the anterior mediastinum and to TETs has been completely revolutionized, making minimally invasive surgery the present “gold standard”, especially for early-stage neoplasms. In this regard, the use of robot-assisted surgical techniques is continuously increasing, given the better surgical field offered and the possibility to articulate the instruments, allowing a precise and adequate dissection. Partial or total sternotomy is, of course, still used, but is reserved for huge masses and advanced cases with invasion of surrounding organs or structures, in particular great vessels [104,105].

The multimodality approach to TETs intends to improve survival and surgical resectability. In advanced/metastatic, recurrent, or inoperable TETs, surgery might be assisted or replaced by radiotherapy and systemic chemotherapy [9]. Postoperative radiotherapy is recommended in thymic carcinoma; on the contrary, postoperative radiation is still debated for radically resected stage II and stage III thymomas. In the case of poor or positive resection margins, postoperative radiotherapy is, however, highly recommended [106,107].

**Figure 3:**
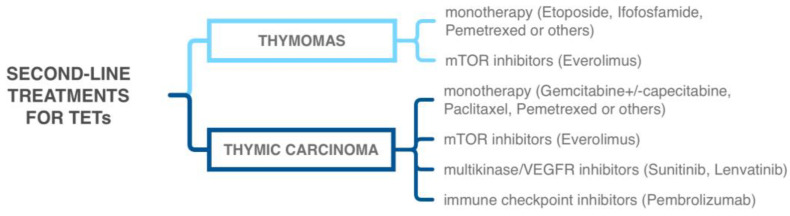
Schematic representation of second-line treatment options for thymic epithelial tumors [9].

Since TETs are deemed to be chemosensitive tumors, platinum-based chemotherapy represents the first-line standard in advanced or unresectable cases. In addition, platinum salts can be combined with etoposide, doxorubicin, cyclophosphamide, paclitaxel, and others. Monotherapy with pemetrexed or ifosfamide is usually employed as a second-line treatment (Figure 3) [9,108]. As previously said, molecular studies paired with the advent of targeted therapies have opened new perspectives in TET treatment, especially in the field of thymic carcinomas.

### 5.1. Tyrosine Kinase Inhibitors

*KIT* and *EGFR* mutations have represented the first targetable alterations explored in TET. Although immunohistochemical expression of c-kit (CD117) is found in up to 80% of thymic carcinomas, it is not paralleled by the presence of the *KIT* mutation, which is found only in up to 11% of cases [109,110,111,112]. When administered to unselected patients with thymic carcinoma or type B3 thymoma with unknown *KIT* mutational status, imatinib showed no effect on thymic carcinomas [113,114,115]. However, in recent years, there have been some reports of mutated cases that responded to imatinib [116,117,118]. Therefore, Schirosi et al. [112] suggest screening of all thymic carcinomas with CD117 immunohistochemistry. In positive cases, especially the ones co-expressing CD5 and p63, *KIT* mutational status must be investigated, as the finding of a *KIT* mutation predicts a potential response to targeted inhibitors.

Unfortunately, *EGFR* mutations are infrequent in TETs and undetectable in thymic carcinomas, so they are not a very suitable therapeutic target. Nonetheless, some aggressive thymomas have shown satisfactory responses to cetuximab [110,119,120]. Again, the increased immunohistochemical expression of ERBB2 (HER2) has been reported in up to 58% of squamous thymic carcinomas [121,122], but an underlying *HER2* amplification is extremely uneventful, thus making HER2-targeted therapy not suitable for TETs [6].

It is known that aberrant angiogenesis has important implications in TET pathogenesis, and several studies have demonstrated the efficacy of VEGF receptor/multikinase inhibitors in TETs. Different trials have tested and proved the efficacy of sunitinib, an anti-angiogenic and multikinase inhibitor targeting VEGF receptors, in thymic carcinomas and thymomas refractory to first-line chemotherapy, reaching disease control in up to 91% of thymic carcinomas and 86% of thymomas. Given this evidence, sunitinib has now been introduced as a standard second-line treatment for patients with thymic carcinoma who have progressed after first-line chemotherapy (Figure 3) [123,124,125,126]. Similarly, lenvatinib, another VEGFR/multikinase inhibitor, showed promising effects on advanced or metastatic thymic carcinomas, with presumably higher efficacy than sunitinib [127]. The RESOUND trial tested successfully regorafenib—a VEGFR, PDGFR, and FGFR inhibitor—as a well-tolerated monotherapy for advanced or recurrent B2 or B3 thymomas and thymic carcinomas [128].

The PI3K/AKT/mTOR pathway is another potential therapeutic target in TETs [129]. As previously discussed, this pathway might be activated by chromosome 19q CNV (mainly in thymomas) or by a mutation in *PTEN*, *PIK3CA*, or *NF1* (in both thymomas and thymic carcinomas) [5,7]. Thus, everolimus, an *mTOR* inhibitor, has been successfully tested in TETs [130,131], but its efficacy is mainly represented by disease stabilization, with limited cases showing a partial or complete response. Furthermore, a considerable percentage of patients (up to 30%) experience severe drug-related adverse events. For these reasons, despite being included as one of the standard second-line therapies for TETs in the NCCN guidelines, some authors suggest only restricting the use of everolimus to selected patients with limited treatment options [9]. Likewise, a clinical trial with buparlisib, a pan-*PI3K* inhibitor, has shown modest activity in B2 and B3 thymomas, with more than half of patients requiring early discontinuation due to dermatologic and pulmonary toxicity, advising extreme caution when approaching the use of *PI3K* inhibitors in TETs [132].

### 5.2. Immune-Checkpoint Inhibitors

In the oncologic field, much attention has been recently paid to the TET microenvironment and potential response to immune-checkpoint inhibitors blocking the PD1/PD-L1 interaction. TMB and PD-L1 expression are the main predictors of responses to immune-checkpoint inhibitors [5]. PD-L1 immunohistochemical expression varies among different TETs, ranging from 13% of type A to 76% of type B3 thymomas, and to 53% of thymic carcinomas [6], and it has been linked to shorter survival [133]. Indeed, many TETs show a “hot” microenvironment, rich in T-cells, B-cells (especially in cases associated with MG), dendritic cells, and tumor-associated macrophages [93]. Additionally, the presence of *CYLD1* mutation might predict a favorable response to PD-L1 inhibitors, while on the contrary, the *BAP1* mutation might correlate with lower PD-L1 expression and response rate to PD-L1 inhibitors [91,134].

So far, three immune-checkpoint inhibitors have been tested in TETs: pembrolizumab, avelumab, and nivolumab [10,11,134,135,136,137]. In seminal work by Giaccone et al., pembrolizumab showed a 22.5% response rate in thymic carcinomas, although up to 15% of patients experienced severe autoimmune toxicity [10]. This study categorized PD-L1 immunohistochemical expression into low (1–49%) or high (≥50%); patients with high PD-L1 expression experienced longer progression-free and overall survival. Nonetheless, patient selection must carefully exclude patients who are experiencing or have experienced autoimmune disease associated with TET to limit potentially fatal drug toxicity [11,138,139]. Similarly, Cho et al. reported an overall response rate of 24.2% in a cohort of 26 thymic carcinomas and 7 thymomas treated with pembrolizumab [140,141]. Despite the preventive exclusion of subjects with active autoimmune diseases, many of the enrolled patients experienced several autoimmune adverse effects (ranging from fatigue to myocarditis) that led to therapy suspension followed by the resolutive administration of corticosteroids.

Avelumab has also shown a promising effect in TETs, either alone or in association with axitinib [136,142]. As regard to combination therapies, three currently ongoing phase II trials are evaluating the putative synergic effects of immune-checkpoint inhibitors and multikinase inhibitors, namely, avelumab plus axitinib and pembrolizumab plus lenvatinib in pre-treated B3 thymomas and thymic carcinomas [135,136], and pembrolizumab plus sunitinib in thymic carcinomas (NCT03463460). Another trial (NCT02364076) is evaluating the association of pembrolizumab with the indoleamine 2–3dioxygenase (IDO)-inhibitor epacadostat. IDO is an enzyme involved in the catabolism of the amino acid tryptophan, playing a significant role in immunosuppression in human cancer [143].

Unlike pembrolizumab and avelumab, nivolumab monotherapy demonstrated substantial inefficacy among both thymomas and thymic carcinomas [144,145]. This notwithstanding, the EORTC NIVOTHYM phase II clinical trial is currently exploring the potential benefit of an nivolumab plus ipilimumab (CTLA-4 inhibitor) association (NCT03134118). Other interesting clinical trials are evaluating an nivolumab association with the VEGFR/PDGFR inhibitor vorolanib in thoracic tumors (NCT03583086) and atezolizumab monotherapy in thymic carcinoma (NCT04321330).

Results of all these clinical trials, of which many are ongoing, will clarify doubts regarding the efficacy, toxicity, and response predictive factors of immune-checkpoint inhibitors, which nonetheless have already entered into clinical practice (Figure 3).

### 5.3. Other Targeted Therapies

Recurrent *CDKN2A* alterations in TETs, mainly due to chromosome 16q loss in thymic carcinoma, as well as the upregulation of *MYC/Max* and *E2F1*, lead to hyperactivation of the CDK4-6/Rb pathway and subsequent cell cycle impairment [5]. Different CDK inhibitors have been tested in TETs, such as milciclib [146] and palbociclib [147], with promising results that need further validation.

Principe et al. [148] also reported an isolated case of a metastatic thymoma with the *BRCA2* mutation that responded well to olaparib, a PARP inhibitor. PARP inhibitors might also be effective in TETs harboring *ATM* mutations, which are, however, extremely rare and only demonstrated in one case of thymic carcinoma [6].

Although epigenetic alterations are frequently observed in thymic carcinomas [88], the efficacy of the histone deacetylase inhibitor drug belinostat has only been modest in TETs, despite its intriguing immunomodulatory effects (such as a decrease in T-regulatory cells) [9,149,150]. The latter might also be additive to immune-checkpoint inhibitor therapy [9,149,150].

Finally, the novel monoclonal antibody radretumab, which targets the extra-domain B fibronectin, a stromal protein, has been tested as radioimmunotherapy in TETs, although with inconsistent results [151].

## 6. Outlooks for the Future

International collaborations are pivotal for rare tumors such as TETs. In recent years, ITMIG provided a new surge in TET translational research, and additional collaborative health networks are still needed to advance this scientific field and turn basic discoveries into more effective treatments [36].

Notably, considerable effort has been expended in defining the molecular landscape of TET. However, molecular diagnostics can currently only support or confirm the histological diagnosis or, in rare cases, identify thymic carcinoma patients with targetable alterations who may benefit from specific inhibitors.

Therefore, ongoing and upcoming trials will further delineate the optimal single medications or treatment combinations for each patient, and the emerging role of targeted therapies and immune-checkpoint inhibitors. Future studies will also better identify the predictive factors for both response and complications.

Moreover, given the histological and molecular diversity of TETs and the lack of a single predictor of biological behavior, it is desirable to develop and use a risk stratification model based on the primary prognostic criteria.

## 7. Conclusions

After almost a century in which novel classifications were published at an impressive rate, the scientific community has finally reached common ground in terms of histological classification, adopting the numbered letter approach as per WHO guidelines, and in terms of staging, narrowing down the choice to either TNM or Masaoka–Koga systems. Except for thymic carcinomas, TETs are largely indolent and slow-growing neoplasms, in which accurate histological subclassification and microscopic evaluation of invasiveness remain the salient features of the diagnostic report, as they drive the biological behavior.

Far from the past turmoil about TET histology and staging, therapeutics now represent the exciting new battlefield for TETs, as demonstrated by the large number of studies and ongoing clinical trials on targeted therapies. TET molecular characterization has been decisive for two main reasons: it has given robust validation to the widely debated histological classification, proving the molecular diversity of different TETs, and it has also represented the genetic blueprint for the application of targeted therapies in TET. Additionally, the study of the TET microenvironment has opened the way for immune-checkpoint inhibitors, which have proven their efficacy either alone or synergically with other targeted therapies. Further studies will continue to expand our knowledge of TET biology and therapy, with the hope of positively impacting the lives of our patients.

## Figures and Tables

**Table 1:** Evolution of the WHO classification of TETs.

1999 WHO Classification (2nd Edition)	2004 WHO Classification (3rd Edition)	2015 WHO Classification (4th Edition)	2021 WHO Classification (5th Edition)
**Thymomas**			
Thymoma, type A Thymoma, type AB Thymoma, type B1 Thymoma, type B2 Thymoma, type B3	Thymoma, NOS Thymoma, type A Thymoma, type AB Thymoma, type B1 Thymoma, type B2 Thymoma, type B3 Micronodular thymoma Metaplastic thymoma Lipofibroadenoma Microscopic thymoma Sclerosing thymomas	Thymoma, NOS Thymoma, type A * Thymoma, type AB Thymoma, type B1 Thymoma, type B2 Thymoma, type B3 Micronodular thymoma with lymphoid stroma Metaplastic thymoma Lipofibroadenoma Microscopic thymoma Sclerosing thymomas	Thymoma, NOS Thymoma, type A * Thymoma, type AB Thymoma, type B1 Thymoma, type B2 Thymoma, type B3 Micronodular thymoma with lymphoid stroma Metaplastic thymoma Lipofibroadenoma
**Carcinomas**			
Thymoma, type C Epidermoid keratinizing (squamous cell) Epidermoid non-keratinizing Lymphoepithelioma-like Sarcomatoid (carcinosarcoma) Clear cell Basaloid Mucoepidermoid Papillary Undifferentiated	Thymic carcinoma Squamous cell Basaloid Mucoepidermoid Lymphoepithelioma-like Sarcomatoid (carcinosarcoma) Clear cell Adenocarcinoma Papillary adenocarcinoma Carcinoma with t(15;19) Undifferentiated	Thymic carcinoma Squamous cell Basaloid Mucoepidermoid Lymphoepithelioma-like Clear cell Sarcomatoid Adenocarcinoma Papillary adenocarcinoma Thymic carcinoma with ACC-like features Mucinous adenocarcinoma Adenocarcinoma NOS NUT carcinoma Undifferentiated carcinoma Combined thymic carcinomas Others	Squamous carcinomas Squamous cell Basaloid Lymphoepithelioma Adenocarcinomas Adenocarcinoma, NOS Low-grade papillary adenocarcinoma Thymic carcinoma with ACC-like features Adenocarcinoma, enteric-type Adenosquamous carcinoma NUT carcinoma Salivary gland-like carcinoma Mucoepidermoid Clear cell Sarcomatoid Carcinosarcoma Undifferentiated carcinomas Thymic carcinoma, NOS
**Thymic neuroendocrine neoplasms**			
Carcinoid tumors (well-differentiated NECs) Classic Spindle cell Pigmented With amyloid (extrathyroidal medullary carcinoma) Atypical LCNEC SCC Mixed small cell-epidermoid keratinizing carcinoma	Carcinoid tumors Typical carcinoid Atypical carcinoid LCNEC SCC Combined TETs, including NEC	Carcinoid tumors Typical carcinoid Atypical carcinoid LCNEC Combined LCNEC SCC Combined SCC	Carcinoid tumor, NOS/NET, NOS Typical carcinoid/NET, grade 1 Atypical carcinoid/NET, grade 2 LLCNEC SCC Combined SCC

* Including atypical variant. Abbreviation: NOS, not otherwise specified; NUT, nuclear protein of testis; TET, thymic epithelial tumor; NEC neuroendocrine carcinoma; LCNEC, large cell neuroendocrine carcinoma; SCC, small cell carcinoma; NET, neuroendocrine tumor.

**Table 2:** TNM staging of TETs, 8th edition (IASLC/ITMIG), and Masaoka–Koga staging.

TNM Staging of TETs, 8th Edition (IASLC/ITMIG)	
Category	Definition
**T descriptor**	
T1a	Encapsulated or unencapsulated, with or without extension into mediastinal fat
T1b	Extension into mediastinal pleura
T2	Direct invasion of the pericardium (partial or full thickness)
T3	Direct invasion of lung, brachiocephalic vein, superior vena cava, chest wall, phrenic nerve, hilar (extrapericardial) pulmonary vessels
T4	Direct invasion of aorta, arch vessels, main pulmonary artery, myocardium, trachea, or esophagus
**N descriptor**	
N0	No nodal involvement
N1	Anterior perithymic nodes
N2	Deep intrathoracic or cervical nodes
**M descriptor**	
M0	No metastatic pleural, pericardial, or distant sites
M1a	Separate pleural or pericardial nodule(s)
M1b	Pulmonary intraparenchymal or distant organ metastasis
**Masaoka–Koga staging**	
Stage I	Grossly and microscopically encapsulated tumor
Stage II a	Microscopic transcapsular invasion
Stage II b	Macroscopic invasion into thymic or surrounding fatty tissue, or grossly adherent but not breaking through mediastinal pleura or pericardium
Stage III	Macroscopic invasion into neighboring organs (i.e., pericardium, great vessels, or lung)
Stage IV a	Pleural or pericardial dissemination
Stage IV b	Lymphatic or hematogenous metastasis

## Data Availability

Not applicable.
